# Heat Hazard Control in High-Temperature Tunnels: Experimental Study of Coupled Cooling with Ventilation and Partial Insulation for Synergistic Geothermal Extraction

**DOI:** 10.3390/ijerph20031941

**Published:** 2023-01-20

**Authors:** Junjian Wang, Zijun Li, Gang Li, Yu Xu

**Affiliations:** 1 School of Resources and Safety Engineering, Central South University, 932 Lu Shan South Road, Changsha 410083, China; 2 Sinosteel Maanshan General Institute of Mining Research Co., Ltd., Maanshan 243000, China

**Keywords:** heat hazard control, partial thermal insulation, ventilation, coupled cooling, geothermal energy extraction

## Abstract

The problem of heat hazard in tunnel engineering has seriously affected the normal work of personnel and machinery. After combining the heat hazard control method of controlling the energy source and blocking the energy transfer, a technical scheme of precise thermal insulation at the working face in concert with geothermal energy extraction is proposed, forming a coupled cooling method of ventilation and partial thermal insulation. By building a scaled model test platform, the temperature field of the working area was analyzed, and the effect of factors, such as with or without a thermal insulation layer, ventilation velocity, and surrounding rock temperature on the cooling limit, was discussed. The feasibility of extracting energy and enhancing cooling through the heat exchange layer was judged. The results show that the partial thermal insulation can effectively weaken the heat dissipation of the surrounding rock and enhance the ventilation and cooling effect, which can reduce the average ventilation limit temperature of the working area by 1.6 °C. The addition of the heat exchange layer can further improve the tunnel environment on the basis of partial insulation, making the cooling limit temperature drop by another 3.1 °C, and the heat exchange layer can work for one year to extract geothermal energy 4.5 × 10^8^ J. The coupled cooling scheme of ventilation and partial thermal insulation is practical and useful, which can provide technical ideas for improving the thermal environment of the tunnel.

## 1. Introduction

With the gradual deepening of mine mining depth [1,2,3] and the construction of high geothermal tunnels in plateau areas guided by the western development plan [4,5,6], heat hazard, a common geological disaster problem, has become a bottleneck restricting the operation of various underground space projects. On the one hand, the high temperature environment will cause huge physical and psychological harm to the staff, such as rapid heart rate [7], fatigue [8], inattention [9] and other adverse reactions, thereby significantly increasing the accident rate. On the other hand, it will also lead to difficulties in the operation of mechanical equipment to dissipate heat, resulting in mechanical aging and a reduction in production efficiency [10]. Therefore, it is urgent to solve the problem of heat hazard in excavation tunnels.

The traditional mine tunnel heat hazard control methods can be divided into two aspects: controlling the energy source and blocking the energy transfer. The main idea of controlling the energy source is to offset the heat source and conduct heat diversion through the cold source, or directly reduce the temperature of the underground heat source. The cold source heat transfer generally adopts the method of ventilation to convey low-temperature air flow to the working area to neutralize and divert the heat emitted by the surrounding rock. Yaping Wang [11] analyzed the effects of different air velocities and air supply temperatures on the high-temperature environment of the tunnel and came up with the optimal air supply conditions that could satisfy the working surface temperature. Qi Chen et al. [12] explored the connection between wind flow velocity and the convective heat transfer coefficient in the construction of tunnel duct air supply, revealing the distribution of the convective heat transfer coefficient at tunnel corners to provide a theoretical basis for optimizing duct ventilation design. Kepeng Wang et al. [13] revealed the thermodynamic characteristics of a deep coal mine as an example and derived a method to calculate the cooling load of a deep tunnel working face. However, due to the long air transport distance in the deep tunnel, the air flow on the ground has a higher temperature when it reaches the working face. In engineering practice, ventilation is usually combined with refrigeration at the same time [14,15,16]. The widespread use of refrigeration equipment imposes a significant economic burden and has problems, such as difficulty in discharging heat from the facility. It is very important to reduce air cooling loss and improve ventilation efficiency to save project costs and improve the working environment quality.

With the gradual strengthening of environmental protection awareness in various countries, heat hazard control methods to directly reduce the temperature of underground heat sources have also been widely studied. As a high-quality clean energy, underground high temperature can reduce the heat dissipation of surrounding rock to the interior of the tunnel when extracted [17,18]. Guozhu Zhang et al. [19] developed a numerical model of the tunnel liner ground heat exchanger and evaluated the effect of ventilation on the thermal performance of the tunnel liner ground heat exchanger. Chao Yang et al. [20] proposed the idea of installing ground source heat pumps into underground tunnels and demonstrated the feasibility of this method to extract geothermal energy by numerical simulation and evaluated its economic benefits. Yu Xu et al. [21] proposed a synergetic mining of geothermal energy in deep mines to control heat hazard and established a fully coupled numerical model to determine that this technique can significantly improve thermal comfort and yield considerable geothermal energy in the tunnel.

The technical idea of blocking energy transfer is to isolate the high-temperature surrounding rock that causes heat hazard from the internal environment of the tunnel. It is a common method to spray thermal insulation material or set up thermal insulation layers on both sides of the tunnel to reduce the heat transfer efficiency. Guozhu Zhang et al. [22] proposed an air-layer structure to insulate the roadway, and the thermal performance of the insulated roadway with air-layer structure was evaluated by numerical simulation, which confirmed that the roadway with air-layer structure has better insulation effects. Fangchao Kang et al. [23] established a heat transfer model for high-temperature tunnels, compared the temperature fields of tunnels with and without insulation, and judged the many factors affecting the insulation effect. Weijing Yao et al. [24] studied the effect of thermal insulation on the radius of the heat transfer circle, the temperature field of surrounding rock, and the wall temperature by the ANSYS numerical simulation method, with sensitivity analysis of thermal insulation with different thermal conductivity and thickness. Nikodem Szlazak et al. [25] insulated the roof and sidewalls, comparing the climatic conditions in excavation tunnels with and without insulation, and the results showed that insulation is essential in high-temperature tunnels. However, there are few construction tests of tunnel insulation. Although the configured materials can play a good role in blocking energy transmission, the overall spraying or laying will greatly increase the process and economic burden in the early stage of project construction.

In previous studies, the engineering scale was usually large, and researchers discussed the influence of the thermal properties of surrounding rock on the overall tunnel heat transfer in a macroscopic way [26,27,28]. However, the influence of the thermal diffusion behavior of surrounding rock in local areas on spatial temperature distribution cannot be ignored [29], but the heat dissipation characteristics of the surrounding rock in excavation tunnels are rarely studied. In our previous research, it was found that the existence of auxiliary ventilation systems in excavation tunnels would bring differences in local thermal diffusion [30,31]. It causes local high temperature in the working area of the excavation tunnel. Therefore, precise thermal insulation and cooling in areas with strong thermal diffusion is proposed as a simple, energy-saving, and efficient heat hazard control method. Based on the study of the thermal diffusion behavior of tunnel surrounding rock, this paper proposes a technical idea of accurate, partial thermal insulation for the working area with the most serious heat hazard and the largest number of workers from a local perspective. By combining the partial heat insulation with the auxiliary ventilation system in the excavation operation, together with the heat exchange system installed on the partial thermal insulation, the purpose of coupled cooling of ventilation and partial thermal insulation for synergistic geothermal energy extraction is realized. The establishment of partial insulation can effectively save the initial construction cost of the project and avoid the waste of materials caused by the overall laying. The precise thermal insulation of the work area can easily achieve a good effect of improving the tunnel environment, reduce the burden of refrigeration required by the ventilation system, and ensure a reasonable allocation of resources. In order to explore the characteristics and heat transfer laws of tunnel thermal environments under partial insulation conditions, a scaled model test platform of coupled cooling of ventilation and partial thermal insulation was built based on the law of similarity. The ventilation and cooling model of excavation tunnels was established by COMSOL Multiphysics, and the correctness of geometric and similar parameters selection in the scaled model test platform was verified. The influence law of partial insulation on the heat transfer of surrounding rock is obtained by analyzing the change of temperature in the tunnel with or without partial insulation. By adjusting the parameters of the ventilation and heat exchange layer, the efficiency of the ventilation coupled cooling and partial insulation was measured and the feasibility of the ventilation coupled cooling and partial insulation in the collaborative geothermal mining working face was judged, which provided a theoretical basis for further optimizing the working environment and saving energy.

## 2. Partial Thermal Insulation Technical Solutions and Advantages

### 2.1. Partial Thermal Insulation Design

In underground space engineering heat hazard, the surrounding rock is the main source of heat [32,33]. In the kilometer-deep mine tunnel, due to poor ventilation and continuous heat exchange between rocks, the local area is in a state of heat balance for a long time, and the initial rock temperature is stable at about 40 °C. Since the rock physical properties of the excavation tunnel in the same deep mine are similar (as shown in Table 1), the heat dissipation of surrounding rock in undeveloped local areas will not be too different.

**Figure 1:**
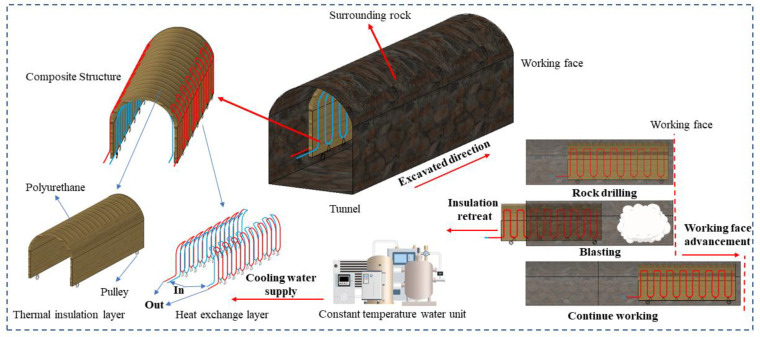
Movable thermal insulation layer structure and partial insulation method.

However, when the tunnel starts to be excavated, the heat dissipation of surrounding rocks in different areas is different. Due to the processes of rock cutting, drilling, and loading explosives, workers have to work 3–6 m away from the working face. In order to ensure the occupational safety of workers, auxiliary ventilation facilities must be added to ensure the cooling and dust removal needs. When ventilation facilities operate, air distribution becomes complicated, and there is a complex heat exchange process among the environment of the excavation tunnel, the high temperature surrounding rock, and the ventilation airflow. In the working area, high air velocity will lead to strong convective heat transfer, resulting in “heat recovery” [30,31,34]. This area is therefore the focus of precise insulation. Figure 1 shows the technical solution for partial insulation of underground space works.

A movable thermal insulation layer (MTIL) is used in this study to achieve partial thermal insulation. Figure 1 shows the construction of a movable thermal insulation layer, which can be spliced into small sections in the design to ensure that the layer is adapted to a wider range of conditions. The heat exchange layer is arranged outside the insulation, using a thermostatic water unit to provide cold water as the medium for heat exchange. In this part, the main objective is to solve the heat hazard problem. The heat extraction aspect is considered when the water that can be provided in the mine allows the heat exchange layer to exchange heat effectively. And when the groundwater cannot meet the cooling requirements, the thermostatic water control device comes into play to provide a lower temperature heat exchange medium to achieve the cooling demands. The heat exchange medium is fed from the inside of the insulation for the first stage of heat transfer with the internal tunnel environment, thus reducing the ambient temperature inside the tunnel. The heat exchange medium is then transferred back to the outside of the insulation layer to reduce heat dissipation from the surrounding rock in the second phase of heat exchange with the surrounding rock, while allowing the heat exchange medium to be transported back to the end of the insulation layer for geothermal energy harvesting at the working face. A number of pulleys are arranged at the bottom of the insulation layer to achieve a movable function of the partial insulation structure, facilitating the pushing with the advance of the working face.

The movable thermal insulation layer is located at the working face with the workers when they are in the tunnel for processes, such as rock drilling and explosive loading. After these processes, the insulation is retreated with the staff to a safe area, and blasting is carried out. After blasting, the insulation continues to move forward with the slag-out process and so on to ensure that the temperature of the personnel working area is always within the allowable range of national standards.

### 2.2. Prospects, Values, and Limitations

The partial insulation discards the previous idea of laying insulation or spraying concrete throughout the tunnel and provides targeted cooling for the working area. From a technical point of view, partial insulation protects the areas where the heat exchange is most intense, blocking the heat dissipated by the surrounding rock while reducing the convective heat exchange between the air flow and the rock wall. The preserved air flow cools the internal environment of the tunnel more. In addition, there is little difference in the effectiveness of partial and total insulation in blocking heat transfer. From an economic point of view, tunnels often extend hundreds or even thousands of meters, so for a single tunnel length of 100 m and commercially available insulating concrete of USD 187.5 per ton, the cost of insulation is approximately USD 30,000, whereas the cost of creating partial insulation is approximately 1 percent of that. Partial insulation not only saves a lot of materials, but also saves workers’ time and effort when laying the whole insulation, which can better speed up the project.

Although the thermal insulation mortar used in the tunnels has a much lower thermal conductivity than ordinary concrete [35,36,37,38], it still falls short of commercially available building insulation materials [39,40]. However, building insulation materials are not suitable for underground space projects due to their high combustibility and low strength [41]. In the case of partial insulation, the insulation covers a small area and does not act as a support, which allows for a preferential selection of materials. The partial insulation can be disassembled and reused many times, even with expensive materials, without increasing costs.

With the gradual expansion of engineering (e.g., the increased depth of mining, the spread of tunnels to the plateau areas, the continuous expansion of the metro, etc.), the geothermal problem is gradually becoming more widespread, and the occupational health of workers is generally at risk. Although the partial insulation solution offers a simple and easy solution to improve the working environment, there are some problems with its implementation. Chief among these problems is the fact that the surrounding rock of the freshly excavated tunnel is not smooth, and the protruding rock is quite disruptive to the movement of partial insulation devices. Therefore, regular tunnels should be insulated with rigid materials according to the actual situation, and irregular tunnels should be designed with insulation curtains made of flexible materials, but the thermal insulation properties of both need further consideration and research. In addition, the partial insulation is not conducive to observing the dynamic changes in the surrounding rock because it does not assume a supporting role and covers a large area. Therefore, it is necessary to install monitoring holes or sensors on the insulation for real-time monitoring. In this study, such reconcilable realities have not been considered, but rather the focus has been on the functionality and efficiency of partial insulation. The neglected elements will be mentioned in the assumptions section.

## 3. Description of Similarity Experiment

When using mathematical methods to study some complex heat transfer processes, realistic or sufficiently accurate solutions are often not obtained because the set conditions are too ideal. Testing directly on the engineering site can have an impact on the productivity of the company, and it is impossible to determine the degree of influence of each factor and to make a single-factor summary of the law. The emergence of similarity theory has filled these gaps by offering the possibility of generalizing individual experimental results to similar phenomena. The following assumptions were made in this paper in order to reasonably simplify the test process:

(1) The rock material is a homogeneous medium and the roughness is ignored.
(2) Ignore the effect of humidity on heat transfer.
(3) Ignore local heat dissipation by personnel and machinery.

### 3.1. Selection of Similarity Parameters

The similarity of the two phenomena presupposes that each element that makes up the model must be similar to its counterpart in the prototype, i.e., the physical field made up of a series of physical quantities must be correspondingly similar. The corresponding results for the prototype can be deduced from the results of the model tests. According to the similarity criterion, when two phenomena are similar, all the dimensionless combinations in the system of equations and singular value conditions of the two phenomena correspond to equal numbers and have the same dimensionless solution [42].

In the test, the heat exchange between the wind flow and the internal environment of the tunnel is mainly considered, so the selection of the similarity index involves fluid mechanics and heat transfer. During tunnel ventilation, the influence of pressure on the flow field is much greater than that of gravity, so the Froude number is ignored, and the main reference is the Reynolds number and the Archimedes number [43,44,45]. The medium in the tunnel where the heat transfer occurs is always air, so that the Prandtl number characterizing the physical properties of the fluid is almost constant [46,47]. In addition, the effects of buoyancy, inertial force, convective heat transfer, and thermal conductivity are also considered, and the Grashof number and Peclet number are analyzed [48,49]. The similarity indexes for fluid mechanics and heat transfer are selected as shown in Table 2.

The main parameters of the scaled model tests are shown in Table 3.

A mathematical model was built with Comsol Multiphysics 6.0 to verify the feasibility of conducting scale model tests using the selected parameters. This study involves a multiphysics coupling of flow and heat transfer, and the governing equations were chosen as follows:

Fluid Mechanics Section:

(1) Mass conservation equation and N-S equation [50,51]:

(1) $$\frac{\partial \rho }{\partial t}+\nabla \cdot \left(\rho u\right)=0,$$

(2) $$\rho \left(\frac{\partial u}{\partial t}+u\cdot \nabla u\right)=-\nabla p+\nabla \left(\mu \left(\nabla u+{\left(\nabla u\right)}^{T}\right)-\frac{2}{3}\mu \left(\nabla \cdot u\right)I\right)+F,$$

where *ρ* is the density of fluid (kg/m^3^), *t* is the time (s), *u* is the velocity vector of fluid (m/s), *p* is the pressure of fluid (Pa), *μ* is the dynamic viscosity (Pa·s), *I* is the unit vector, and *F* is the momentum source term (kg/(m^3^·s)).

(2) Realizable k-ε turbulence equation [52]:

(3) $$\rho \frac{\partial k}{\partial t}+\rho u\cdot \nabla k=\nabla \cdot \left(\left(\mu +\frac{\mu_{t}}{\sigma_{k}}\right)\nabla k\right)+P_{k}+P_{b}-\rho \varepsilon ,$$

(4) $$\rho \frac{\partial \varepsilon }{\partial t}+\rho u\cdot \nabla \varepsilon =\nabla \cdot \left(\left(\mu +\frac{\mu_{t}}{\sigma_{\varepsilon }}\right)\nabla \varepsilon \right)+C_{\varepsilon 1}\frac{\varepsilon }{k}P_{k}-C_{\varepsilon 2}\rho \frac{\varepsilon^{2}}{k},$$

where *C_ε1_, C_ε2_, σ_k_*, and *σ_ε_* are realizable k-ε constants. *C_μ_* is related to the mean strain rate and is defined by Equation (5):

(5) $$C_{\mu }=\frac{1}{A_{0}+A_{S}\frac{U^{*}k}{\varepsilon }},$$

where *A_0_*, *A_s_*, and U* are functions of velocity gradients.

Heat transfer section:

(1) Heat transfer within the rock [53]:

(6) $$\rho C_{P}\frac{\partial T_{S}}{\partial t}+\rho C_{P}u\cdot \nabla T_{S}=\nabla \cdot \left(K_{S}\Delta T_{S}\right)+\left(\nabla \cdot q\right)+Q,$$

where *C_p_* is the specific heat capacity, J/(kg·K); *T_S_* is the temperature of the solid, K; *K_S_* is the thermal conductivity of the solid, W/(m·K); and *Q* is the heat resource, W/m^3^.

(2) Heat transfer between fluids [54]:

(7) $$\rho C_{P}\frac{\partial T_{g}}{\partial t}+\rho C_{P}u\cdot \nabla T_{g}=\alpha_{P}\left(\frac{\partial p_{A}}{\partial t}+u\cdot \nabla p_{A}\right)-\left(\nabla \cdot q\right)+\tau :S+\nabla \cdot \left(K_{g}\Delta T_{g}\right)+Q,$$

where *α_P_* is the coefficient of thermal expansion, 1/K; *T_g_* is the temperature of air, K; *P_A_* is the absolute pressure, Pa; *τ* is the viscous stress tensor, Pa; *S* is the strain rate tensor, 1/s; and *K_g_* is the thermal conductivity of gas, W/(m·K).

(3) Heat exchange at fluid-solid coupling interfaces [55]:

(8) $$q_{c}=h_{k}(T_{g}-T_{s})+h_{c}(T_{g}-T_{s}),$$

where *q_c_* is the heat flux by conduction and convection, W/m^2^, and *h_k_* and *h_c_* are the heat conductivity coefficient and convective heat transfer coefficient, respectively, W/(m^2^·K).

To obtain a mesh-independent solution for the simulation results, the grid independence verification was carried out, and the results are shown in Table 4. As the number of grids increases, both the air temperature and ventilation volume tend to stabilize at the grid number of 38,000. Taking into account the accuracy of the solution and the speed of the calculation, the grid number was chosen to be 43,072.

Forty monitoring locations were evenly selected at the central axis of the tunnel, and the temperature errors between the model and the prototype are shown in Table 5.

The relative error is calculated by the following formula:

(9) $$RE=\frac{P_{v}-M_{v}}{P_{v}}\times 100\%,$$

where *RE* is the relative error, %; *P_v_* is the calculated value of the prototype, K; and *M_v_* is the calculated value of the model, K.

As can be seen from Table 3, the relative error between the model and the prototype does not exceed 0.015%, with a maximum value of −0.014%. Errors of this magnitude are negligible in engineering, so it can be assumed that the parameters were chosen correctly and that the tests carried out in the model can provide a reference for discussing the phenomenon in the engineering prototype.

### 3.2. Composition of the Test Platform

The built test platform for the coupled cooling of ventilation and partial insulation scale model consists of three main components: the ventilation unit, the data acquisition unit, and the temperature control unit. The ventilation unit consists of the axial fan, tapering tube, reducer, ventilation ducts, and model tunnel; the data acquisition unit consists of the KEYSIGHT DAQ970A Data Acquisition System, Pt100 thermocouples, and Fluke 923 air velocity meter; and the temperature control unit consists of the electric heating belts, temperature controller, and insulation cotton for the tunnel exterior.

#### 3.2.1. Test Platform Dimensions and Layout

**Figure 2:**
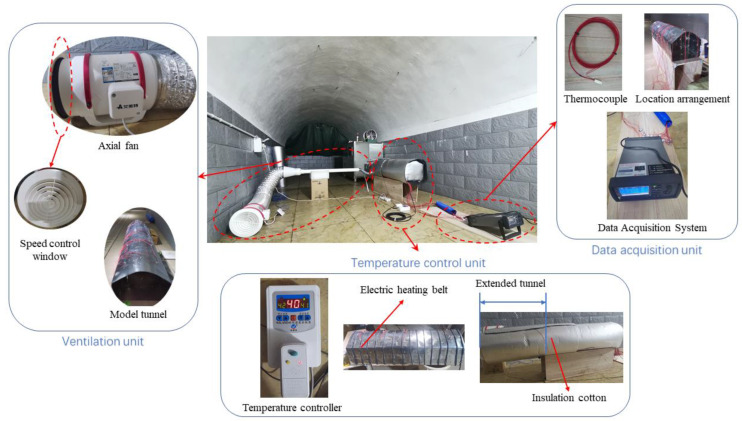
Scale model test platform for coupled cooling of ventilation and partial thermal insulation.

The prototype referenced for the construction of the test platform is a conventional arch shape. The scaled model tunnel width is 0.3 m, the sides of the surrounding rock are 0.18 m high, and the arch height is 0.28 m. The tunnel monitoring area is the part of the length of 1 m from the working face. In addition, to prevent the influence of the tunnel exit boundary conditions on the monitoring area, the tunnel exit was extended to create a virtual tunnel up to 2 m. The ventilation duct has a diameter of 0.05 m and is suspended 0.025 m from the floor. The test model is heated by wrapping an electric heating belt evenly around the exterior of the surrounding rock and adjusting it to the required temperature by means of a thermostat. Wrap insulation cotton around the outside of the electric heating belt to ensure the stability of the temperature control and to avoid excessive errors caused by fluctuations in the external ambient temperature. A number of thermocouples were placed inside the model tunnel, connected to the data acquisition system for real-time temperature monitoring, and the input air flow velocity was controlled in the ventilation system by means of air windows. The layout of the test platform is shown in Figure 2.

#### 3.2.2. Monitoring Scheme

**Figure 3:**
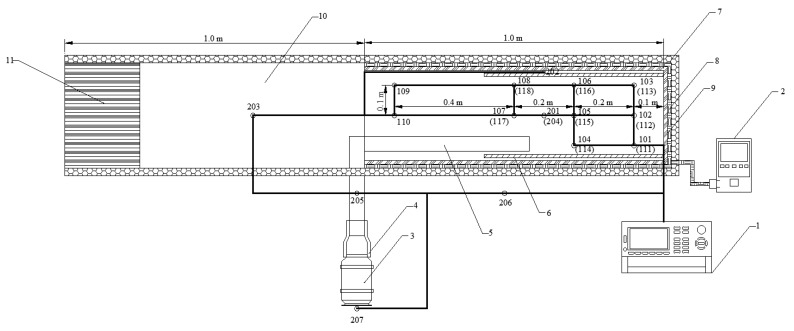
Distribution of temperature-monitoring locations on the test platform. 1—Data acquisition system; 2—Temperature controller; 3—Axial fan; 4—Reducer; 5—Ventilation duct; 6—Partial thermal insulation baffle; 7—Insulation cotton; 8—Electric heating belt; 9—Surrounding rock; 10—Virtual tunnel; 11—Tunnel movable exit.

A total of 24 temperature-monitoring locations were set up on the test platform, including 18 temperature-monitoring locations for working areas (101–118) and 3 monitoring locations for surrounding rock walls (201-roof, 202-left wall, and 204-floor). The remaining 4 monitoring locations were the inlet of the fan (207), the extended tunnel (203), the outlet of the ventilation duct (205), and the test environment (206). The distribution of each temperature-monitoring location is shown in Figure 3.

Monitoring locations 101–108 are at a height of 0.2 m above the floor, and monitoring locations 109–118 are at a height of 0.1 m above the floor. The data scan interval for all monitoring locations is 1 s.

### 3.3. Test Strategy

The experiment investigates the effect of the presence or absence of insulation in a ventilation tunnel on the high-temperature environment and determines the feasibility and cooling efficiency of partial insulation devices. The presence or absence of insulation, ventilation time, air velocity, and surrounding rock temperature were used as study variables in the tests, with temperature variation in the tunnel as the target variable for analysis. In addition, extraneous variables, such as heating method, fan heat dissipation, and ambient temperature, were kept as consistent as possible to avoid any influence on the test results. Table 6 depicts the ambient temperature (including pre-test) at each test.

Before starting the experiment, set the velocity of the duct outlet to the required value and fix the position of the duct. Due to the different positions of the thermocouples and the fact that the heating of the surrounding rock is not absolutely uniform, the ambient temperature control in the tunnel is based on the monitoring point with the slowest temperature rise reaching the required value. In addition, the temperature controller is equipped with sensors arranged on the tunnel rock wall to control the heating efficiency and keep the temperature of the surrounding rock within a certain range to ensure the accuracy of the experiment.

## 4. Analysis of the Temperature Field in the Partial Insulated Tunnel

### 4.1. Partial Thermal Insulation Effect

**Figure 4:**
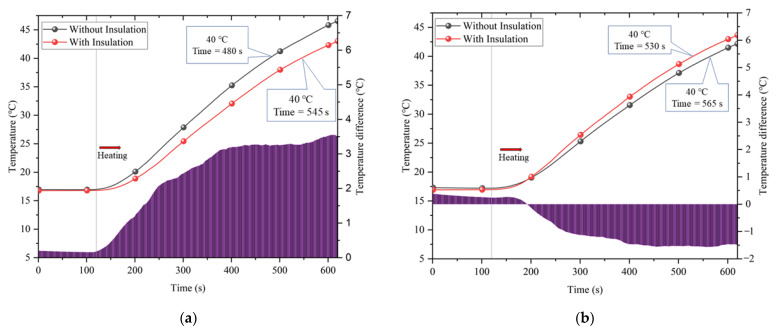
Comparison of partial insulation effects: (**a**) Covered area; (**b**) Uncovered area.

To investigate the effect of partial insulation, heating tests were carried out in a tunnel with and without insulation. In this paper, the insulation was chosen to be made of polyurethane, in a length of 0.6 m, placed close to the working face and 0.01 m from the sides of the tunnel. Heating started 120 s after the data acquisition was started, and the heating process continued for 500 s. The results are shown in Figure 4, with data collected at monitoring location 101 in the insulation-covered area and location 203 in the uncovered area.

Figure 4a shows that the tunnel with the insulation layer heated up more slowly. For example, it took 545 s for a tunnel with insulation and only 480 s for a tunnel without insulation to reach 40 °C. This means that tunnels with insulation in the project will reach high temperatures almost 2 h later. The temperature difference with and without insulation is shown at the bottom of Figure 4a. At the beginning of the heating, the temperature difference was small, but as the heating continued, the temperature difference gradually increased and eventually tended to stabilize, with a final stable temperature difference of approximately 3.5 °C. Figure 4b shows that the opposite situation exists in the uncovered area as opposed to the covered area, where the temperature is higher in the uncovered area of the tunnel with partial insulation. This is due to the presence of the insulation affecting the direction of heat dissipation, with more heat escaping towards the rear of the tunnel along the gap between the insulation and the surrounding rock. After accounting, the average temperature of the working area of the tunnel without insulation reached 45.0 °C when heated continuously for 600 s, while the average temperature of the working area of the tunnel with insulation was only 41.6 °C.

### 4.2. Temperature Field Distribution of Partially Insulated Tunnel

**Figure 5:**
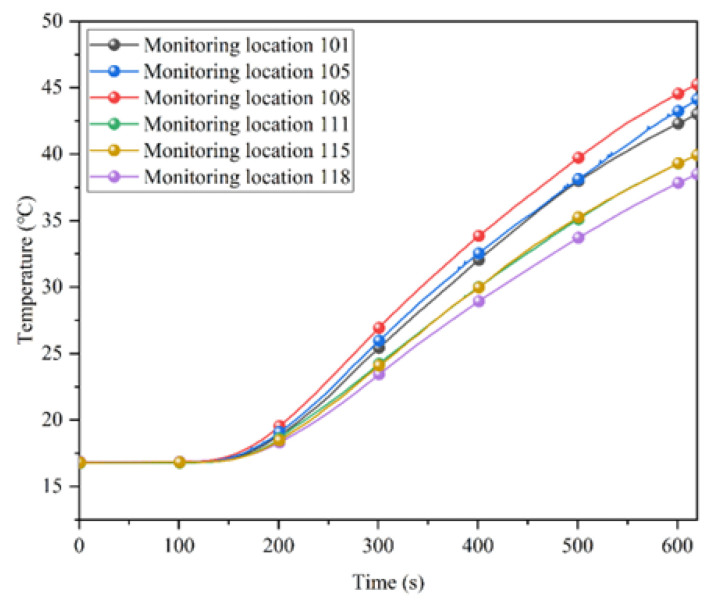
Partially insulated tunnel monitoring-point temperatures.

Due to differences in the position of the monitoring locations, the temperature at each location varies. Figure 5 shows the variation in temperature at the monitoring locations in the partially insulated tunnel.

**Figure 6:**
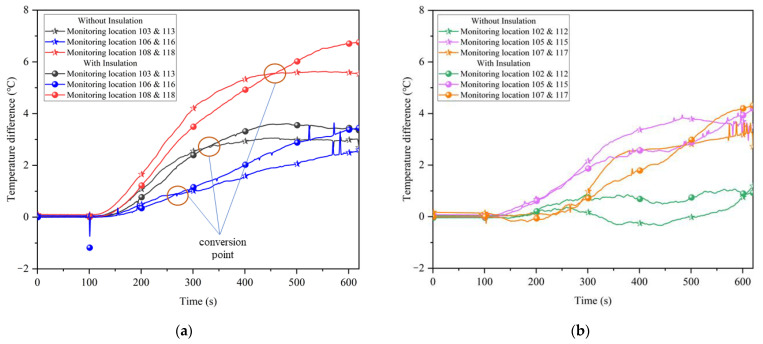
Temperature difference between vertical sections at selected monitoring locations: (**a**) Left side of tunnel; (**b**) Middle of tunnel.

In Figure 5, it can be seen that the upper monitoring locations (i.e., monitoring locations 101–108) had higher temperature than the lower monitoring locations (i.e., monitoring locations 109–118). There are two main reasons for such difference: on the one hand, the heating time is short, and the heating is carried out continuously, the density of the air decreases as the temperature rises, and the hot air moves upwards to accumulate; on the other hand, the tunnel is arched, and the area of the top plate is much larger than the bottom plate, which has a larger area for heat dissipation, and the temperature is higher at the monitoring points near the top plate. The above phenomenon is not related to the placement of partial insulation, which has an effect on the temperature field distribution mainly on the temperature difference between the upper and lower monitoring locations. The temperature difference between vertical sections at some of the monitoring locations is shown in Figure 6.

Figure 6a shows that on the left side of the tunnel, the temperature difference between the upper and lower monitoring locations gradually shows an increasing trend with increasing heating time. In the early stages of heating, the different heat dissipation areas of the surrounding rock walls, and the fact that the walls reached similar temperature within a short period of time, resulted in a large difference in temperature between the upper and lower parts of the tunnel without insulation, while the tunnel with insulation had no significant difference in temperature between the upper and lower parts because the temperature diffusion was slower. After heating for a period of time, the conversion point was reached. As the upper monitoring locations were closer to the roof of the tunnel, they were more severely affected by heat dissipation, and the heat from the rock wall on both sides would spread upwards through the gap between the insulation and the surrounding rock, while the lower monitoring locations were located inside the insulation and were only affected by heat dissipation from the floor, so the insulation effect gradually manifested. The temperature difference between the upper and lower parts of the tunnel with insulation rises and exceeds that of the tunnel without insulation.

The conversion points represent the times when the insulation affects the change in temperature between the upper and lower monitoring locations, where monitoring locations 106 and 116 were located in the middle of the insulation and the conversion points arrived earlier. Monitoring locations 103 and 113 were slow to reach the conversion point due to their proximity to the working face and the effect of heat dissipation from the working face. Monitoring locations 108 and 118 were located at the back end of the insulation, where the insulation was weakened, thus reaching the conversion point the latest. Figure 6b shows the variation of the temperature difference between the upper and lower part of the tunnel at the central axis, which does not show the regular variation as in Figure 6a because the temperature at the center was less influenced by the insulation. The unevenness of the temperature of the surrounding rock in the test results is a diffusion of heat in the tunnel that is not linear. According to the second law of thermodynamics, the temperature at the center of the tunnel should develop towards a certain stable value. With insulation, the temperature difference between the center and rear monitoring locations (i.e., 105 and 115, 107 and 117) was much lower than without insulation. This means that the insulation makes the temperature spread more evenly in the center of the tunnel. The monitoring locations at 102 and 112 were close to the working face and were heavily influenced by heat dissipation, resulting in a small temperature difference between the top and bottom, close to 0.

Combined with what is shown in Figure 4b, the general distribution of the temperature field under partial insulation can be derived. Due to the existence of gaps between the insulation layer and the surrounding rock, most of the heat is dissipated from above and behind the gaps, thus presenting a phenomenon that the upper temperature is greater than the lower temperature, and the front temperature difference is lower than the rear temperature difference in the partially insulated tunnel.

## 5. Analysis of the Coupling Effect of Ventilation and Partial Thermal Insulation

### 5.1. Effect on Cooling Limit

**Figure 7:**
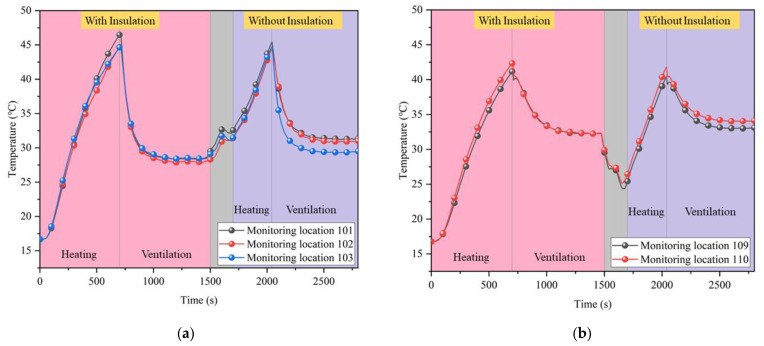
Temperature variation in the tunnel under coupled cooling test: (**a**) Inside the insulation area; (**b**) Outside the insulation area.

The following tests were carried out to investigate the effect of with or without partial insulation on the ventilation cooling limit. First, the virtual tunnel outlet was closed and heating was started. When the overall ambient temperature inside the tunnel was above 40 °C, the outlet was opened, and ventilation was started for a duration of 800 s. After this period, the heating and ventilation were stopped, and the insulation was removed. The heating and ventilation were repeated as described above. Using a model inlet air velocity of 7.5 m/s as an example, the temperature variation curve over time was obtained for some of the monitoring locations in the tunnel, as shown in Figure 7.

The first half of Figure 7a,b is both with insulation, which was removed after 1500 s of testing (i.e., the central grey area), and the second half is tested without insulation. Figure 7a shows that in the condition with partial insulation, the monitoring point reached a lower temperature at steady state than without insulation. This is because the insulation layer blocks the direct contact between the airflow and the high-temperature surrounding rock, reducing the loss of airflow cooling during convective heat transfer. In Figure 7b, with or without insulation had no significant effect on the cooling limit because the positions of the monitoring locations were outside the partial insulation. This also confirms that convective heat transfer between the airflow and the surrounding rock is not significant at the rear of the air shaft outlet.

**Figure 8:**
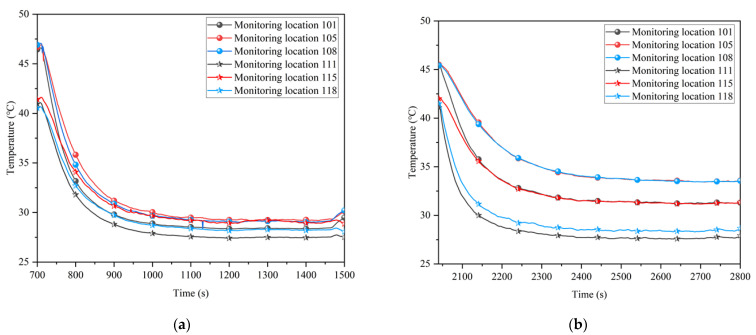
Comparison of cooling limits at upper and lower monitoring locations with and without insulation: (**a**) With insulation; (**b**) Without insulation.

In the case of partial insulation, the upper and lower monitoring locations end up with similar cooling limits, whereas without insulation, the lower monitoring locations near the ducts can be reduced to lower temperature. The cooling limits for the upper and lower monitoring locations with and without insulation are shown in Figure 8.

As the air flow was only convective heat exchange with the working surface when there was insulation, more of the cooler volume was used to cool the tunnel environment, resulting in a lower overall temperature. Conversely, when there was no thermal insulation, the cooler air flow was used to offset the convective heat exchange with the surrounding rock and thus failed to cool the upper tunnel environment. The average temperature at each monitoring location in the partial insulation range was calculated to be 29.6 °C with insulation and 31.2 °C without insulation. The difference is 1.6 °C, while the difference in temperature limit at some monitoring locations is much higher than this value, up to 4.3 °C. Combined with the rate of temperature rise in the tunnel, this results in a cooling savings of approximately 2.6 × 10^6^ J per day when using coupled cooling of partial insulation and ventilation.

### 5.2. Effect of Ventilation Speed

**Figure 9:**
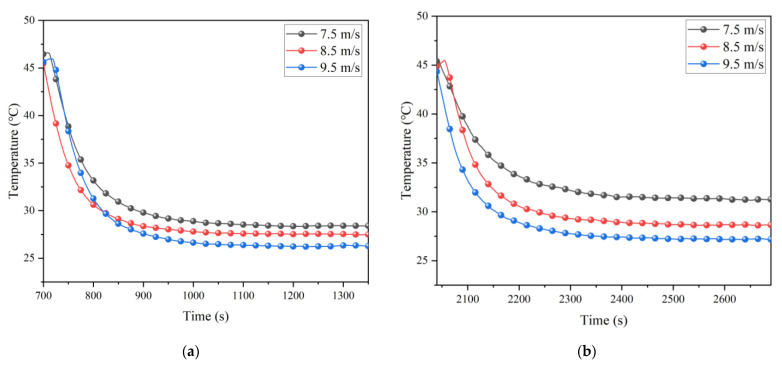
Average cool-down at different air velocities with and without insulation: (**a**) With insulation; (**b**) Without insulation.

In order to explore the influence of air velocity on the partial insulation effect, the model air velocity was adjusted to 7.5 m/s, 8.5 m/s, and 9.5 m/s for experiments. To ensure that the ambient temperature was consistent in the test, the same steps as those in Section 5.1 were used, and the results are shown in Figure 9.

Figure 9 shows that the achievable cooling limit decreased with increasing air velocity and was more pronounced when without insulation. This indicates that the partial insulation effect decreased with increasing air velocity for the same initial temperature and rock temperature. The cooling effect at 8.5 m/s air velocity with insulation in the figure is almost equal to the cooling effect at 9.5 m/s air velocity without insulation. Therefore, when the minimum dust removal air velocity is met, the practical engineering can reduce the consumption of air volume by providing partial insulation.

### 5.3. Effect of Surrounding Rock Temperature

**Figure 10:**
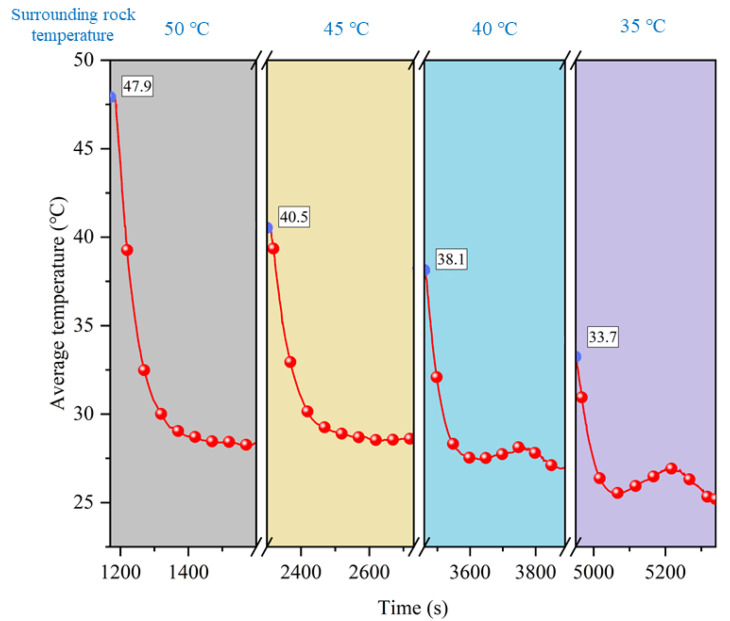
Cooling effect at different surrounding rock temperatures.

The temperature of the surrounding rock is one of the main factors affecting ventilation and cooling. The effect of the initial temperature of the surrounding rock on the coupling of ventilation and partial thermal insulation was investigated by controlling the temperature of the surrounding rock at 50, 45, 40, and 35 °C. The corresponding ambient temperatures in the tunnel were 47.9, 40.5, 38.1, and 33.7 °C, respectively. In this paper, the top plate temperature was used as a reference for temperature control, and the test results are shown in Figure 10.

The effect of coupled ventilation and partial insulation for different rock temperatures is illustrated in Figure 10, where it can be seen that the cooling effect increases as the rock temperature decreases. This effect is reflected in two ways. Firstly, the tunnel with a low surrounding rock temperature can reach a lower temperature after cooling for the same ventilation conditions. Secondly, at the start of ventilation, the temperature of a tunnel with a low rock temperature drops more quickly, and the cooling limit can be reached in a shorter period of time.

### 5.4. Effect of Single and Double Heat Exchange Layer on Cooling Effect

**Figure 11:**
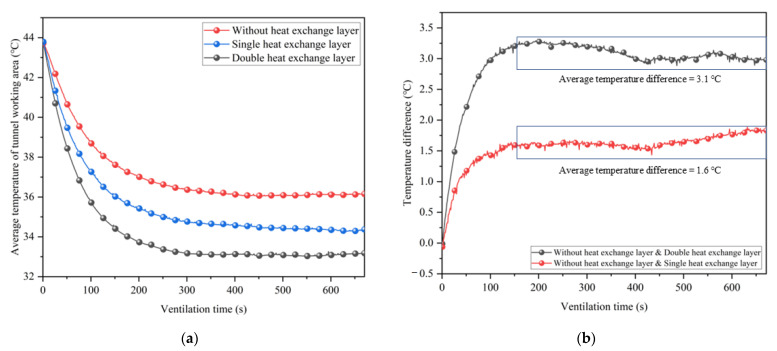
Comparison of the average temperature in the working area of different types of heat exchange layers: (**a**) Average temperature; (**b**) Temperature difference.

In this section, a heat exchange layer is attached to the original partial insulation layer to realize the coupled cooling of ventilation and partial insulation for synergistic geothermal extraction. In order to better reflect the working effect of the heat exchange layer, the inlet air temperature in this section is set higher than in the previous test, with an average of 26.6 °C. The ventilation speed is set to a constant 8.5 m/s. The average temperature variation with ventilation time in the working area of the tunnel with a single heat exchanger layer (the heat exchanger layer is only arranged inside the insulation layer), a double heat exchanger layer (the heat exchanger layer is arranged on both sides of the insulation layer), and without a heat exchanger layer is shown in Figure 11.

**Figure 12:**
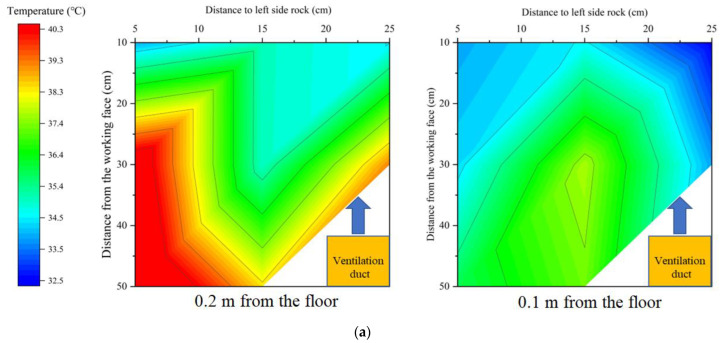

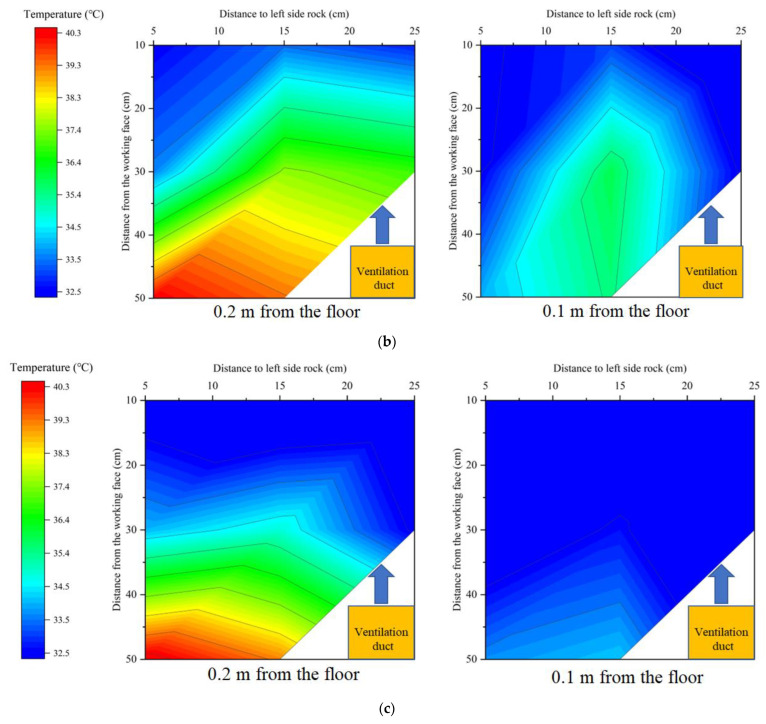
Working area temperature field under different heat exchange layer arrangement cases: (**a**) Without heat exchange layer; (**b**) Single heat exchange layer; (**c**) Double heat exchange layer.

In Figure 11, the average temperature in the working area of the tunnel is highest when reaching thermal equilibrium without the additional heat exchange layer. The placement of the heat exchanger layer can effectively reduce the tunnel temperature further. From Figure 11b, it can be seen that a single heat exchange layer can reduce the thermal equilibrium temperature in the tunnel by 1.6 °C on average, and a double heat exchange layer can reduce the thermal equilibrium temperature in the tunnel by 3.1 °C. Despite the fact that the second heat exchange layer is installed on the outside of the insulation, the cooling effect is nearly doubled. To investigate the effect of single and double heat transfer layers on the temperature field of the working area in the tunnel, the equilibrium temperature of the working area in the tunnel under each case was organized into a cloud diagram, as shown in Figure 12.

**Figure 13:**
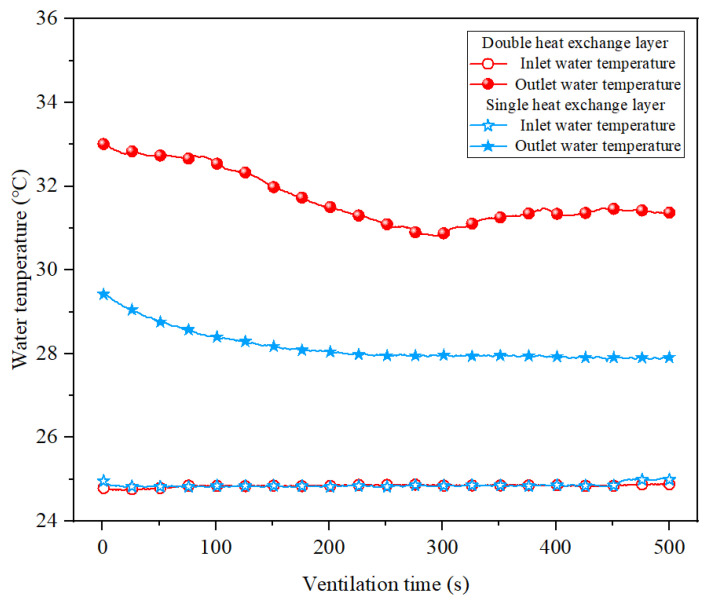
Variation of inlet outlet water temperature under different heat exchange layer cases.

As can be seen in Figure 12, the temperature in both the upper and lower parts of the working area decrease with the addition of heat transfer layers and the increase in the number of layers. The difference is that in the lower part of the working area, the shape of the temperature field does not change, and the area of high temperature is fixed in each case. Since the auxiliary ventilation ducts are installed in the lower part of the tunnel, the air velocity below the tunnel is larger, and the coupling effect is formed between the fresh air flow and the heat exchange layer, so the temperature field distribution is closely related to the trajectory of the air flow. The upper part of the tunnel is more directly affected by the heat exchange layer, and the low-temperature heat exchange medium comes in contact with the hot air in the tunnel to lower the temperature. In general, the existence of the heat transfer layer can significantly improve the efficiency of the coupled cooling of ventilation and partial thermal insulation, while the cooling effect of the double heat exchange layer is much higher than that of the single heat exchange layer. Adding a heat exchange layer on the basis of partial thermal insulation can promote the further improvement of the heat environment in the tunnel. In addition, considering the purpose of synergistic geothermal energy extraction, the temperatures of the input and output water were monitored, and the results obtained are shown in Figure 13.

Figure 13 depicts a large difference in water temperature at the inlet and outlet of the heat exchanger layer, indicating that geothermal energy extraction can be achieved by attaching a heat exchanger layer to the partial insulation. In the initial stage of ventilation, the temperature of the collected return water is higher, which is due to the warming of the accumulated water in the heat exchange layer with the heating of the tunnel during the heating stage, resulting in a larger amount of heat energy extraction in the initial stage, while it gradually tends to stabilize afterwards. During the measurement, the heat energy extraction of the double heat exchange layer is higher, with an average temperature difference of 6.9 °C between the inlet and outlet water, while the single layer can reach only 3.3 °C. Combine the flow rate of the medium in the experimental heat exchange layer with a similar theory, for example, a single heat exchange layer can extract geothermal energy of 2.151 × 10^8^ J and a double layer can extract geothermal energy of 4.5 × 10^8^ J with one year of operation.

### 5.5. Effect of Heat Exchanger Pipe Arrangement Density on Cooling Effect

**Figure 14:**
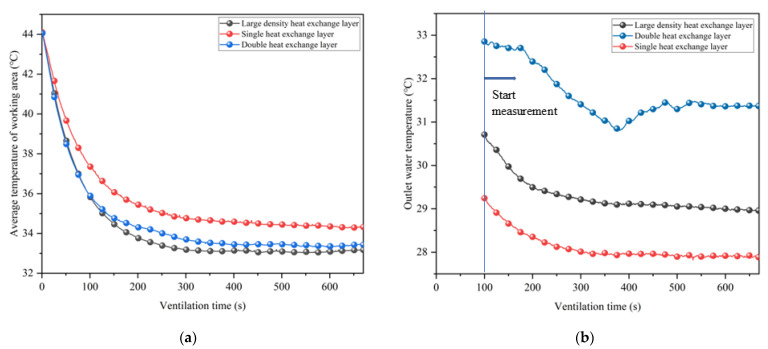
Average temperature of working area and water at different heat exchange pipe density: (**a**) Average temperature; (**b**) Water temperature.

In addition to the number of heat transfer layers, considering that the density of heat transfer pipes arranged on one side of the insulation layer may also be an important parameter affecting the cooling and heat extraction effect, the following test was done: a denser heat transfer pipe was arranged on one side of the insulation layer, so that the length of the heat transfer pipe was the same as the total length of the pipes arranged on both sides in the double heat transfer layer for the cooling test. The average temperature of the working area and the temperature of the water extracted from the heat exchange layers are shown in Figure 14.

In Figure 14a, the final cooling effect after increasing the density of the heat exchange pipes is similar to the setting of a double heat exchange layer. This indicates that it is mainly the effective heat transfer length that affects the heat transfer effect. However, in Figure 14b, the heat extraction effect of the double heat transfer layer is much higher than that of the large density heat transfer layer. This is due to the fact that one side of the double heat transfer layer is almost next to the surrounding rock, and the heat transfer effect is obvious while reducing the heat dissipation from the surrounding rock, so more heat can be extracted while achieving almost the same cooling effect. Therefore, when wanting to enhance the cooling effect by adjusting the heat exchange layer, the density of the heat exchange pipe arrangement should be increased, and if wanting to extract more geothermal energy, double insulation should be adopted to obtain more economic benefits without increasing additional pumping power.

## 6. Conclusions

Aiming at the heat hazard problem in underground tunnels, this paper proposed a new technical idea of coupled cooling of ventilation and partial insulation. Based on the similarity principle, the geometric parameters and similar parameters were determined and calculated, and the experimental platform of the ventilation and partial insulation coupling cooling scale model was established. The three-dimensional ventilation and cooling model of tunnel flow and heat transfer coupling was established by COMSOL, and the rationality of similar parameter selection was verified. By adjusting the parameters and modes of heat insulation, ventilation, and heat transfer, the feasibility of the coupling method of ventilation and local heat insulation and cooling is verified by experiments. The main conclusions are as follows:

(1) The concept of coupled cooling of ventilation and partial thermal insulation is proposed. The precise insulation of the working area can effectively enhance the ventilation cooling effect and improve the tunnel environment, while saving insulation materials and reducing the unnecessary investment in the tunnel construction.
(2) By constructing a test platform for coupled cooling of ventilation and thermal insulation, it was confirmed that the construction of partial insulation can improve the high-temperature environment in the working area. The construction of partial insulation makes the temperature field in the tunnel appear higher at the top than at the bottom and the difference lower at the front than at the back. When ventilation and partial heat insulation are coupled to reduce temperature, the partial thermal insulation layer can make the ventilation cooling limit decrease. The average temperature of the working area is reduced by 1.6 °C.
(3) The ventilation velocity and the temperature of the surrounding rock are important factors affecting the coupled cooling effect of ventilation and partial thermal insulation. As the air velocity increases, the cooling effect gradually increases, and the thermal insulation effect gradually decreases. As the temperature of the surrounding rock increases, the efficiency of the coupled cooling of ventilation and partial thermal insulation decreases.
(4) The further addition of a heat exchange layer on top of the insulation layer can achieve a better cooling effect and realize the synergistic exploitation of geothermal energy. The cooling effect is proportional to the length of the heat exchanger tube, and the best heat energy extraction effect is achieved by the double heat insulation layer, which can extract about 4.5 × 10^8^ J of heat energy in 1 year of operation.

## Figures and Tables

**Table 1:** Thermal parameters of rocks.

Parameters	Value
Specific heat capacity of rock (J/(kg·°C))	1300
Heat conduction coefficient of rock (W/(m·°C))	3.5
Density of rock (kg/m^3^)	2600
The initial temperature of rock (°C)	40–45

**Table 2:** Similarity index for fluid mechanics and heat transfer.

Similarity Index	Representation
Reynolds number	Reflecting the ratio of inertial to viscous forces
Archimedes number	Reflecting the ratio of buoyancy to inertia force
Grashof number	Reflecting the ratio of buoyancy to viscous force
Prandtl number	Reflecting the relationship between momentum transfer and heat transfer

**Table 3:** Scale model test parameters.

Parameter	Symbols	Scale	Relations
Length	*C_l_ = l_m_:l_p_*	1:10	*-*
Volume	*C_v_ = v_m_:v_p_*	1:1000	*C_v_ = C_l_^3^*
Velocity	*C_u_ = u_m_:u_p_*	10:1	*-*
Time	*C_t_ = t_m_:t_p_*	1:100	*C_t_ = C_l_/C_v_*
Density	*C_ρ_ = ρ_m_:ρ_p_*	1:1	*-*
Viscosity	*C_μ_ = μ_m_:μ_p_*	1:1	*-*
Convective heat transfer coefficient	*C_h_ = h_m_:h_p_*	10:1	*C_h_ = C_λ_/C_l_*
Thermal conductivity	*C_λ_ = λ_m_:λ_p_*	1:1	*-*
Specific heat	*C_cp_ = Cp_m_:Cp_p_*	1:1	*-*
Temperature	*C_T_ = T_m_:T_p_*	1:1	*-*

**Table 4:** Grid Independence Verification.

Grid Number	Air Temperature (°C)	Ventilation Volume (m^3^/min)
1124	25.48	21.87
1476	25.11	21.63
1723	25.79	21.63
2106	25.52	21.47
2733	25.66	21.36
5585	24.35	23.11
13,102	24.34	23.61
38,100	26.70	27.05
43,072	26.72	27.17

**Table 5:** Prototype and model relative error.

Monitoring Location Number	Relative Error (%)	Monitoring Location Number	Relative Error (%)
1	2.62 × 10^−4^	21	−1.22 × 10^−2^
2	−8.70 × 10^−4^	22	−1.22 × 10^−2^
3	−1.75 × 10^−3^	23	−1.24 × 10^−2^
4	−2.74 × 10^−3^	24	−1.24 × 10^−2^
5	−3.71 × 10^−3^	25	−1.25 × 10^−2^
6	−4.67 × 10^−3^	26	−1.26 × 10^−2^
7	−5.53 × 10^−3^	27	−1.32 × 10^−2^
8	−6.44 × 10^−3^	28	−1.36 × 10^−2^
9	−7.18 × 10^−3^	29	−1.33 × 10^−2^
10	−8.09 × 10^−3^	30	−1.34 × 10^−2^
11	−8.75 × 10^−3^	31	−1.35 × 10^−2^
12	−9.60 × 10^−3^	32	−1.27 × 10^−2^
13	−1.01 × 10^−2^	33	−1.18 × 10^−2^
14	−1.06 × 10^−2^	34	−1.19 × 10^−2^
15	−1.10 × 10^−2^	35	−1.36 × 10^−2^
16	−1.12 × 10^−2^	36	−1.45 × 10^−2^
17	−1.14 × 10^−2^	37	−1.42 × 10^−2^
18	−1.16 × 10^−2^	38	−1.39 × 10^−2^
19	−1.18 × 10^−2^	39	−1.36 × 10^−2^
20	−1.20 × 10^−2^	40	−1.20 × 10^−2^

**Table 6:** Temperature variation in the test ambient.

Test Number	1	2	3	4	5	6	7	8	9	10	11	12	13
Ambient temperature (°C)	16.5	16.5	16.6	16.6	16.5	17.1	16.7	16.7	17.0	16.5	16.2	16.4	16.5

## Data Availability

Not applicable.
